# The validity and precision of the leicester cough questionnaire in COPD patients with chronic cough

**DOI:** 10.1186/1477-7525-10-4

**Published:** 2012-01-09

**Authors:** Farida F Berkhof, Lisenka N Boom, Nynke E ten Hertog, Steven M Uil, Huib AM Kerstjens, Jan WK van den Berg

**Affiliations:** 1 Isala klinieken, department of pulmonary diseases, Zwolle, the Netherlands; 2 University Medical Center Groningen, department of pulmonary diseases, and University of Groningen, Groningen, the Netherlands

**Keywords:** LCQ, COPD, validity, cough, health status

## Abstract

**Background:**

A validated instrument to assess the effects of chronic cough on health status in patients with chronic obstructive pulmonary disease (COPD) is currently not available. The Leicester Cough Questionnaire (LCQ) is a cough-specific health status questionnaire which is originally validated for a population of general patients presenting with chronic cough. We examined the psychometric performance of the LCQ in patients with COPD and chronic productive cough.

**Methods:**

Concurrent validity, internal consistency, reproducibility and responsiveness were determined. The St. George's Respiratory Questionnaire (SGRQ) and the Short Form-36 (SF-36) were used as external criteria. Questionnaires were completed at the start of the study. After 2 and 12 weeks the LCQ was repeated, together with a global rating of change.

**Results:**

In total 54 patients were included. Concurrent validity analysis showed significant correlations between corresponding domains of the LCQ and the SGRQ (r_s _-0.31 to -0.60). Corresponding domains of the LCQ and the SF-36 showed weaker correlations (r_s _0.04 to 0.41). Internal consistency was adequate for two of the three domains (Cronbach's α 0.74 - 0.86). Test-retest reliability in stable patients was high (intraclass correlation coefficients 0.79 - 0.93). The mean difference after two weeks was 0.73 (± 1.75). Responsiveness analysis indicated that the LCQ was able to detect changes after 12 weeks.

**Conclusion:**

The LCQ is a valid, reliable, responsive instrument to measure health status in COPD patients with chronic productive cough.

**Trial Registration:**

ClinicalTrials.gov: NCT01071161

## Background

COPD is a leading cause of morbidity and mortality all over the world. In 2001 COPD was the fifth cause of death and its relative importance is predicted to increase in future years [1,2]. Detection of airflow limitation is paramount in the GOLD definition of COPD [3,4], but clinically COPD is characterized by chronic and progressive dyspnea, cough and sputum production [4,5]. Prevalence rates of chronic productive cough in the male COPD population are estimated to be 15-44% and 6-17% in females. These rates increase with age and are strongly related to smoking [6]. The high prevalence of cough in COPD may be caused by increased production of mucus, by the inability to produce a sufficiently large expiratory flow leading to ineffective clearing of the mucus, and by impaired mucociliary clearance leading to mucus retention. Also, many patients with COPD have bronchiectasis [7,8].

Chronic productive cough in COPD patients is associated with severe exacerbations which require hospitalization [9]. These exacerbations have serious effects on health status and quality of life [10].

Improving health status in COPD patients is a management goal in the GOLD guideline. To measure this, assessment is recommended on regularly basis [4]. Although cough is a frequent symptom in COPD, the impact of cough on health status in these patients is largely unknown [2]. One study found that only a small part (2%) of the variance in the scores of the St. George's Respiratory Questionnaire (SGRQ), a disease-specific health status questionnaire is explained by cough [2]. Several cough-specific health status questionnaires have been developed and validated in the general population presenting with cough but not necessarily with COPD [11-15]. Of these questionnaires only the Leicester Cough Questionnaire (LCQ) [16] is available in Dutch [17]. Thus, well validated cough-specific health status questionnaires for COPD patients are absent, rendering it impossible to evaluate patients health status both individually and in clinical trials.

In this paper we investigated the precision and validity of the LCQ in COPD patients with chronic cough.

## Methods

### Study design

The study was designed as a prospective validation study. Blinded data were used from a larger clinical trial in which the effects of azithromycin on cough related health status were studied. Patients were randomised between azithromycin and placebo for twelve weeks and started at day 1. The study was registered at ClinicalTrials.gov (NCT01071161) and was approved by the Ethics Committee of the Isala klinieken, Zwolle, Netherlands (NL19886.075.07, local number: 07.0971). All questionnaires were administered during the first visit, the LCQ was repeated after two and twelve weeks.

### Subjects

Patients with COPD (GOLD II-IV) who were ≥ 40 years of age were eligible to participate if they were suffering from chronic productive cough, defined as productive cough for at least three months a year, in two subsequent years. The inclusion period lasted from September 2009 to September 2010. Exclusion criteria were: a prior history of asthma, use of intravenous or oral corticosteroids and/or antibiotics for an exacerbation three weeks before inclusion, suffering from other relevant lung or liver diseases at the discretion of the treating physician, pregnancy or lactation, use of macrolides in the last six weeks prior to inclusion, allergy or intolerance to macrolides, or other study medication started two months prior to inclusion.

### Questionnaires

The LCQ is a cough-specific health status questionnaire that is well validated in the general population. It consists of 19 items which are divided over 3 domains: physical, psychological and social. A 7-point Likert scale is used to rate. It assesses the impact of cough over the preceding 2 weeks. The total score ranges from 3-21; a higher score corresponds to a better health status [16,18,19]. We have previously described the validation of the Dutch translation for the general population [17].

The St. George's respiratory questionnaire (SGRQ) is a disease-specific health status questionnaire for asthma and COPD, which assesses the impact of symptoms over the preceding 3 months. It contains 76 items divided in 3 sections: symptoms, activity and impacts. The scores range from 0-100, a low score indicates a good health status [20,21].

The Short Form Health Survey (SF-36) questionnaire is a self administrated generic health status questionnaire containing 36 items that cover 9 health dimensions. The SF-36 comprises 8 health scales: physical functioning, role limitations physical, bodily pain, general health, vitality, social functioning, role limitations emotional, and mental health. One single item is used to assess any change in health. Each dimension is scaled from 0-100, higher scores represent better health status [22-26].

A global rating of change (GRC) was used to evaluate self-perceived health change on a 15 point scale (-7 a very great deal worse, 0 no change, +7 a very great deal better).

### Validity & Precision

The following concepts were assessed to determine psychometric performance of the LCQ in COPD: concurrent validity, internal consistency, reproducibility, responsiveness and floor or ceiling effects.

Concurrent validity (appropriate correlations between established measures and the new questionnaire) was measured with the SGRQ and SF-36 [16]. Ideally, we would have used an additional cough-specific questionnaire. However, such questionnaires have not been specifically developed for, nor tested in COPD patients [11-15]. We used the SGRQ as the reference standard.

Internal consistency concerns the degree to which scores of items in a questionnaire correlate homogeneously, and was assessed using data from the LCQ of the first visit.

Reproducibility is a measure of precision and concerns the degree to which repeated measurements in a stable persons (defined as GRC = -1, 0 and 1 in our study) correspond. Reproducibility can be divided in agreement and reliability [27]. Agreement concerns the closeness of the results of repeated measurements after two weeks and assessment is preferred if the aim is to measure change in health status, whereas reliability denotes the degree to which patients can be distinguished from each other, despite measurement error [28]. Both parameters were obtained by comparing the LCQ scores of week 0 and week 2.

Responsiveness is the ability to detect important within-patient changes, even if they are small; it was determined by comparing the LCQ scores of the first visit with LCQ scores after 12 weeks in patients who perceived a significantly improvement in cough symptoms (arbitrarily chosen as GRC≥4 (moderately better to a very great deal better) in our study.

Furthermore the floor or ceiling effects can be assessed if more than 15% of the patients achieve the lowest or highest possible score, respectively. Absence of floor or ceiling effects indicates a good content validity [17,27].

### Statistical Analyses

The concurrent validity was determined by calculating correlation coefficients between LCQ-scores and scores on SGRQ and SF-36. Depending on the distribution of the variables Pearson correlation coefficients or Spearman rank correlation coefficients were used. We made a priori assumptions of the associations between the LCQ total and domain scores and the corresponding scores of the SGRQ and SF-36, respectively. We expected correlation coefficients ≥0.5 for associations between the LCQ and SGRQ and ≥0.4 between the LCQ and SF-36. Corresponding domains of the LCQ physical domain were the SGRQ activity and symptoms domains, and for the LCQ psychological and social domains the SGRQ impact domain [20]. For the LCQ physical domain, the corresponding domain of the SF-36 were the physical functioning/role physical domains, and for the LCQ psychological domain the SF-36 mental health domain and for the LCQ social domain the SF-36 social functioning domain [16].

Internal consistency of the LCQ was evaluated using Cronbach's alpha coefficients for the three domains and the total LCQ. Cronbach's alpha coefficients between 0.7 and 0.9 are considered as proof of internal consistency. Agreement over time was assessed by constructing a Bland-Altman plot for the LCQ total score [29]. Reliability was analysed by calculating Intraclass Correlation Coefficients (ICC) for the 3 domains and the total LCQ [17].

Responsiveness was measured as the area under the receiver operating characteristic (ROC) curve which indicates the probability of correctly identifying subjects who report improvement [27,30].

Data analyses were performed using the Statistical Package for the Social Sciences (SPSS) version 18.0 (SSPS, Chicago, IL, USA).

## Results

### Patients

Fifty-four patients met the inclusion criteria. All patients were eligible in the cross-sectional analyses (concurrent validity, internal consistency, floor or ceiling effects). Data from 52 patients could be used for reproducibility analysis. Data from 49 patients were used to test responsiveness. Two patients withdrew the informed consent after one week. One patient stopped after 4 weeks because of chronic diarrhoea. Two patients failed to return the questionnaire after 12 weeks. Baseline characteristics are shown in table 1 and 2. Most of the patients were male and current smokers with moderate to severe COPD.

**Table 1:** Patient characteristics

n		54
Sex, male, n (%)		40 (74)
Age (years), mean (SD)		68 ± 10
Pack-years, mean (SD)		36 ± 22
Current smoker, n (%)		22 (41)
FEV_1 _(litres)*		1.3 ± 0.5
FEV_1 _% predicted		47 ± 13
COPD GOLD, n (%)†	II	27 (50)
	III	19 (35)
	IV	8 (15)
Respiratory medication, n (%)	Inhaled corticosteroids	51 (94)
	Short acting bronchodilator‡	2 (4)
	Long acting bronchodilator§	35 (65)
	Both ║	17 (31)

* Forced Expiratory Volume in one second

† Classification by the global initiative for Chronic Obstructive Lung Disease [3]

‡ Short acting bronchodilator: short acting beta-2 agonist and/or short acting anticholinergic

§ Long acting bronchodilator: long acting beta-2 agonist and/or long acting anticholinergic

║ Both long and short acting beta-2 agonist and/or anticholinergic bronchodilators

**Table 2:** Baseline health status scores

		mean (SD)	Range
LCQ*	physical	4.2 (± 0.8)	(1.8 - 5.8)
	psychological	4.8 (± 1.0)	(2.3 - 6.6)
	social	4.6 (± 1.3)	(1.0 - 6.5)
	total	13.6 (± 2.8)	(5.9 - 18.1)

SGRQ†	symptoms	65.3 (± 17.4)	(26.7 - 92.8)
	activity	66.2 (± 24.3)	(0 - 100)
	impact	39.9 (± 18.9)	(1.6 - 77.3)
	total	52.1 (± 18.5)	(5.9 - 81.2)

SF-36‡	physical functioning	36.1 (± 25.2)	(0 - 90)
	role physical	23.1 (± 35.6)	(0 - 100)
	pain	61.6 (± 25.6)	(22 - 100)
	general health	32.6 (± 19.0)	(0 - 75)
	vitality	47.1 (± 18.3)	(15 - 90)
	social functioning	65.0 (± 27.1)	(0 - 100)
	role emotional	72.0 (± 37.5)	(0 - 100)
	mental health	69.6 (± 18.7)	(24 - 100)

* Leicester Cough Questionnaire

† St. George's Respiratory Questionnaire, a disease-specific health status questionnaire

‡ Short form 36, a generic health status questionnaire

### Concurrent Validity

Since most of the distributions were skewed, Spearman rank correlation coefficients were used. The correlation coefficients are summarized in table 3. The concurrent validity showed significant correlations between the corresponding domains (described in the statistical analysis section) of the LCQ and the SGRQ. Only the correlation between the psychological domain of the LCQ and the corresponding impact domain of the SGRQ was low to moderate and did not meet the pre-defined minimal level of 0.50.

**Table 3:** Concurrent validity

	LCQ*			
	Physical	Psychological	Social	Total
**SGRQ**†				
Symptoms	-0.57 (54; < 0.001)	-0.45 (54; 0.001)	-0.51 (53; < 0.001)	-0.58 (53; < 0.001)
Activity	-0.58 (54; < 0.001)	-0.11 (54; 0.42)	-0.39 (53; 0.004)	-0.42 (53; 0.002)
Impact	-0.67 (54; < 0.001)	-0.31 (54; 0.023)	-0.60 (53; < 0.001)	-0.61 (53; < 0.001)
Total	-0.68 (54; < 0.001)	-0.28 (54; 0.037)	-0.57 (53; < 0.001)	-0.60 (53; < 0.001)

**SF-36**‡				
Physical functioning	0.39 (54; 0.004)	0.06 (54; 0.65)	0.29 (53; 0.039)	0.28 (53; 0.041)
Role physical	0.31 (53; 0.022)	0.05 (53; 0.72)	0.23 (52; 0.099)	0.22 (52; 0.11)
Pain	0.47 (53; < 0.001)	0.29 (53; 0.038)	0.47 (52; 0.001)	0.47 (52; < 0.001)
General health	0.42 (54; 0.002)	0.25 (54; 0.072)	0.36 (53; 0.008)	0.37 (53; 0.007)
Vitality	0.64 (54; < 0.001)	0.24 (54; 0.086)	0.49 (53; < 0.001)	0.50 (53; < 0.001)
Social functioning	0.41 (54; 0.002)	0.32 (54; 0.017)	0.41 (53; 0.002)	0.43 (53; 0.001)
Role emotional	0.05 (53; 0.70)	0.04 (53; 0.77)	0.13 (52; 0.38)	0.10 (52; 0.48)
Mental health	0.40 (54; 0.003)	0.30 (54; 0.026)	0.37 (53; 0.006)	0.44 (53; 0.001)

Spearman correlation coefficients are presented (n; p-value).

* Leicester Cough Questionnaire

† St. George's Respiratory Questionnaire, a disease-specific health status questionnaire

‡ Short form 36, a generic health status questionnaire

Correlation coefficients for the LCQ and most of the corresponding domains of the SF-36 were low, and almost non existent for the psychological domain. Except the correlations between the social domain of the LCQ and the social functioning domain of the SF-36 (r = 0.41; p = 0.002).

### Internal consistency

The internal consistency of the LCQ, shown in table 4 in the column on the right, was adequate (≥ 0.70) for two of the three domains and the total questionnaire, with Cronbach's alpha coefficients ranging from 0.74 to 0.86. For the physical domain the Cronbach's alpha coefficient was 0.67. The results were comparable with the studies by Birring and Huisman in the more general population presenting with cough but not necessarily with COPD (table 4) [16,17].

**Table 4:** Internal consistency

	Cronbach's alpha coefficient		
LCQ*	**Birring**[16]**†**	**Huisman**[17]**†**	**This study‡**
physical	0.79	0.77	0.67
psychological	0.89	0.84	0.75
social	0.85	0.83	0.74
total	0.92	0.93	0.86

* Leicester Cough questionnaire

† patients with chronic cough

‡ patients with COPD and chronic productive cough

### Reproducibility

**Figure 1:**
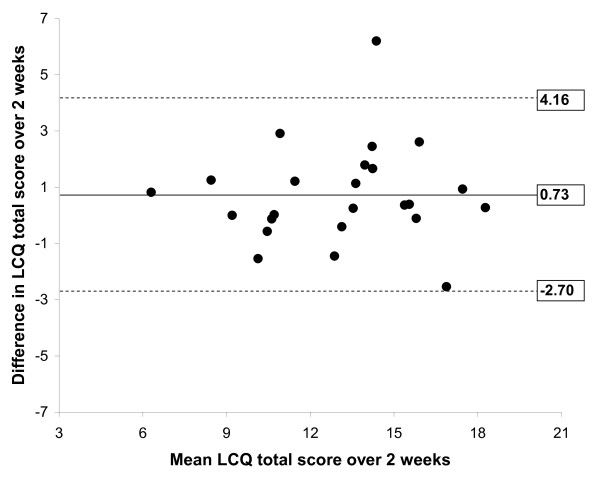
**Bland-Altman plot of LCQ total score repeated over 2 weeks in stable patients representing agreement**. The mean difference over 2 weeks is represented by the solid line. The dashed lines are the limits of agreement, which represent 2 times the standard deviation of the mean difference.

Reproducibility was tested in 24 stable patients. The ICC's for the LCQ are shown in table 5. Except for the psychological domain all repeated measurements were highly correlated, which indicates high test-retest reliability. A Bland-Altman plot of the LCQ total score is shown in Figure 1. The mean difference after two weeks was 0.73 (± 1.75). The upper limit of agreement for the LCQ total score is 4.16 and the lower limit of agreement -2.70.

**Table 5:** Reliability

	Intraclass correlation coefficient			95%CI*
**LCQ†**	**Birring**[16]**‡**	**Huisman**[17]**‡**	**This study§**	
Physical	0.93	0.86	0.93	0.84; 0.97
Psychological	0.90	0.93	0.79	0.51; 0.91
Social	0.88	0.93	0.88	0.72; 0.95
Total	0.96	0.93	0.92	0.81; 0.96

* 95% Confidence Interval for ICC this study

† Leicester Cough Questionnaire

‡ patients with chronic cough

§ patients with COPD and chronic productive cough

### Responsiveness

**Figure 2:**
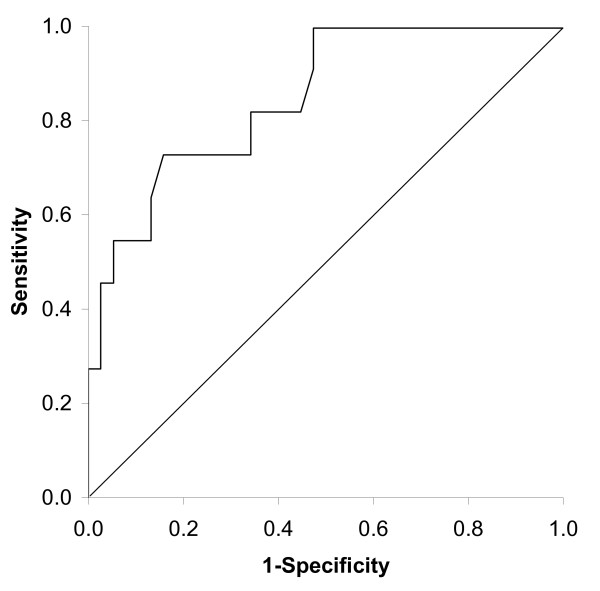
**Receiver Operating Curve for responsiveness of the LCQ total score in COPD patients with chronic cough (n = 49)**. The Area Under the Curve is 0.85 (95%CI: 0.73; 0.97).

Eleven of the forty-nine patients perceived a significant improvement in cough (GRC≥4). In these patients the mean change in the total LCQ score after 12 weeks was 4.3 ± 2.5. The Area Under the Curve (AUC) of the ROC was 0.85 (95%CI 0.73; 0.97, p < 0.001, n = 49), Figure 2. According to Terwee et al, an AUC of > 0.70 is considered to be adequate [27]. Thus, the LCQ was able to detect changes in this specific group of patients.

### Floor or ceiling effect

Floor or ceiling effects (the worst or the best possible score) were analysed at baseline, table 2. Only one patient (1.9%) had the worst possible score in the social domain of the LCQ. No best possible scores were found. Thus, floor or ceiling effects were not present, both in the domains and in the total questionnaire.

## Discussion

This study is the first to examine the validity and precision of the LCQ specifically in COPD patients with chronic productive cough. It shows that the LCQ in these patients reliably measures the same construct as the original LCQ in patients with chronic cough in the general population. Responsiveness analysis indicated that the change in LCQ total scores after 12 weeks was able to predict which patients reported improved health status and which did not. No floor or ceiling effects were present which assured good content validity.

Good concurrent validity of the LCQ was found in relation to the SGRQ but not with the SF-36. This may be explained by both questionnaires measuring different concepts, but more importantly, this is caused by the nature of these questionnaires: the LCQ measures symptom-specific health status and the SGRQ COPD-specific health status, while the SF-36 measures generic health status. The results regarding concurrent validity were in accordance with Birring's original validation study but slightly lower compared to the Dutch validation of the LCQ [16].

In general, the LCQ had an acceptable internal consistency, supporting the hypothesis that the associated questionnaire items are related to each other but do not completely overlap in which case the Cronbach's alpha would have a value of 1, and the item (or domain) would be redundant. The exception with poorer internal consistency was the physical domain (Cronbach's alpha coefficient = 0.67). Three items contributed most to the lower Cronbach's alpha in the physical domain: loss of energy, hoarseness and smoking (questions 9, 14 and 15). As most patients with COPD have a smoking history and many suffer form loss of energy or hoarseness these three items may be less discriminative in COPD patients than in patients with chronic cough. When these items were removed from this domain, the Cronbach's alpha coefficient increased to almost 0.70.

Previous studies showed comparable Cronbach's alpha coefficients which varied between 0.77 and 0.91, except for the physical domain [16,17].

To examine reproducibility, test-retest reliability and agreement were assessed after two weeks in clinically stable patients. Total score and scores on all domains were repeatable with intraclass correlation coefficients above 0.7. So, repeatability of the LCQ in COPD patients with chronic cough was adequate and in accordance with previous results [16,17].

Agreement was assessed in clinically stable patients after 2 weeks according to the method described by Bland and Altman [29]. We found a mean change in the LCQ total score of 0.73 (± 1.75), similar to the results of Birring (0.73 (± 0.94)) after two weeks [16]. Agreement is regarded as acceptable when the limits of agreement are smaller than the minimal clinical important difference (MCID). In previous studies the MCID for the LCQ total score in patients with chronic cough was estimated between 1.3 (± 2.3) and 2.8 (± 2.0) [31,32]., In this study we found limits of agreement above these values, indicating inadequate agreement. However, we realise that this randomised controlled trial was not the ideal setting to measure reproducibility specifically since patients received either active (azithromycin) or placebo medication from the first day. This treatment/placebo difference will have increased noise, resulting in larger limits of agreement and rendering assessment of reproducibility poorer. To draw a more definitive conclusion this analysis should therefore be repeated in clinically stable COPD patients not receiving any (or stable) study medication. The MCID specifically for COPD patients with chronic cough should also be obtained.

The LCQ has been validated and used primarily in patients suffering from chronic cough but not exclusively. Murray et al validated the LCQ in patients with bronchiectasis. They concluded that the questionnaire was able to measure quality of life for assessing existing and new therapies [19]. Both concurrent validity, reliability and responsiveness were comparable with our results. Polley et al undertook a cross-sectional comparison of the LCQ and the Cough specific Quality of life Questionnaire (CQLQ) in patients with either chronic cough, bronchiectasis, COPD or asthma. The group of COPD patients was small (n = 18), but had similar baseline LCQ scores as our participants. They demonstrated significant concurrent validity (r = -0.49) for the total score of the LCQ and the CQLQ in COPD patients. Remarkably, we found a better concurrent validity (r = -0.60) when using the SGRQ, which is not a cough-specific questionnaire. Like in our study, the psychological domain in their study showed weaker correlations in COPD patients than in patients with chronic cough. They reasoned that chronic cough is associated with more psychological problems in women than in men. In both studies the majority of patients included were male, in Polley et al 83% and in our study 74%. Possibly the relatively weak correlations can be explained by this gender imbalance. An additional explanation, they suggest, is that in COPD patients physical complaints are more prevalent than in chronic cough patients, in which psychosocial complaints are predominant [33].

There are some limitations to our study. First it is based on participants in the setting of a randomised double-blind controlled trial. During the analyses of this validity study it was unknown which participants were treated with antibiotics and which were not. In case of significant treatment effect, this will, as earlier mentioned, have influenced reproducibility. Secondly, to assess internal consistency Cronbach's alpha coefficients as well as factor analysis is recommended. The latter analysis was not done in our study, because another method of item reduction was used during the development of the LCQ [16]. Furthermore, we realise that it is difficult to confirm factor structures in different populations [34]. And last, the SGRQ was used as the reference standard. A recent study showed that the SGRQ measures health status only partly. It concluded that the SGRQ can be used mainly for measuring subjective symptoms and impairments and that other aspects of health status such as physical activity, dyspnoea, fatigue or quality of life in general are covered less. Preferably, different questionnaires should be combined [35].

Ideally, questionnaires which measure health status should be both discriminative (able to distinguish patients with different degrees of disease severity) and evaluative (able to detect within patients changes following therapy). In this study the emphasis is mostly on the evaluative properties of the LCQ, because the main goal of the study was to validate the LCQ for use in clinical trials. The discriminative properties should be assessed in a future study.

In summary, our study shows that the LCQ can be used in COPD patients to measure cough-related health status. This provides a tool to study the antitussive or mucolytic effects of drugs in patients with COPD and chronic productive cough.

## Competing interests

The authors declare that they have no competing interests.

## Authors' contributions

FFB contributed to data collection, analysis and interpretation of the data and was primarily responsible for writing the manuscript, LNB and NEH contributed to data collection, analysis and interpretation of the data and critical revision of the manuscript. SMU contributed to the design of the study, analysis and interpretation of the data and critical revision of the manuscript. HAMK and JWKB contributed to the design of the study, interpretation of the data and critical revision of the manuscript. All authors read and approved the final manuscript.
